# Navigating the Complexities: Addressing the Challenges of Aneurysmal Atrial Septal Defects

**DOI:** 10.7759/cureus.59030

**Published:** 2024-04-25

**Authors:** Gade Sandeep, Subrata K Singha, Jitendra V Kalbande, Anil Gupta

**Affiliations:** 1 Anaesthesiology, All India Institute of Medical Sciences, Raipur, Raipur, IND; 2 Anaesthesiology and Critical Care, All India Institute of Medical Sciences, Raipur, Raipur, IND; 3 Cardiac Anaesthesiology, All India Institute of Medical Sciences, Raipur, Raipur, IND

**Keywords:** arrhythmias, cardiogenic embolism, transesophageal echocardiography, transthoracic echocardiography, atrial septal defect, aneurysmal

## Abstract

Aneurysmal atrial septal defects (ASDs) represent a rare subset of congenital cardiac anomalies, characterized by bulging of the interatrial septum. This condition poses unique challenges in diagnosis, management, and outcomes due to its variable clinical presentation and associated complications. While echocardiography remains the cornerstone of diagnosis, advanced imaging modalities such as cardiac magnetic resonance imaging (MRI) and computed tomography (CT) may provide additional insights. Optimal management strategies for aneurysmal ASDs require careful consideration of patient-specific factors, including the size and location of the defect, associated cardiovascular abnormalities, and the presence of pulmonary hypertension. Surgical repair, whether through conventional open-heart techniques or transcatheter interventions, remains the primary treatment modality; however, the approach may vary based on individual patient characteristics. Anesthetic considerations, including hemodynamic monitoring and perioperative care, are crucial in optimizing outcomes and reducing the risk of complications during surgical interventions. Long-term follow-up is essential to monitor potential complications such as residual shunting, arrhythmias, and the development of pulmonary vascular disease. Collaborative efforts among cardiologists, cardiothoracic surgeons, anesthesiologists, and other multidisciplinary specialists are paramount in providing comprehensive care for patients with aneurysmal ASDs.

## Introduction

An atrial septal defect (ASD) occurs in about 25% of children and is among the most common types of congenital heart diseases. It includes defects that involve the true septal membrane and those that allow communication between both atria [1]. Aneurysmal ASDs refer to an abnormal localized bulging or enlargement of the atrial septum, with an incidence of 1-2.5% in the general population [2], while typical ASDs involve a straightforward, uniform defect in the interatrial septum without any associated aneurysm formation [3]. The management of patients with aneurysmal ASDs poses challenges as it requires careful observation and earlier intervention to mitigate complications such as cardioembolic stroke. Understanding the nature and implications of this condition is crucial for effective diagnosis and management. Here, we describe a case of a 36-year-old female who was diagnosed with aneurysmal ASD and planned for surgical repair.

## Case presentation

A 36-year-old female weighing 53 kg presented with complaints of shortness of breath (NYHA grade 3) and palpitations over the past three months. The patient did not have any history of orthopnea, paroxysmal nocturnal dyspnea, or cerebrovascular accidents. On physical examination, a grade 2/6 pansystolic murmur was heard at the left upper sternal border. Transthoracic echocardiography (TTE) revealed an aneurysmal ASD with multiple fenestrations (images not available). Color flow Doppler showed the presence of a left-to-right shunt (L→R). The right atrium and right ventricle were dilated. Tricuspid annular plane systolic excursion (TAPSE) was 17 mm, and the main pulmonary artery was dilated. The left ventricle size and contractility were normal with an ejection fraction of 65%.

Device closure was not attempted in this case due to the difficulty in deploying the device across the bulging interatrial septum. Furthermore, due to the multiple fenestrations, it was anticipated that there could be a residual shunt. The patient was thereby referred to the cardiovascular thoracic team for further evaluation and surgical repair.

The patient’s preoperative hematological and biochemical investigations were within normal limits. She was cleared for surgery under the American Society of Anesthesiologists Physical Status (ASA-PS) class III and was advised to take routine premedications: Tab. alprazolam 0.25 mg, Tab. pantoprazole 40 mg, and a carbohydrate drink two hours before the surgery as per the Enhanced Recovery After Surgery (ERAS) protocol.

**Figure 1:**
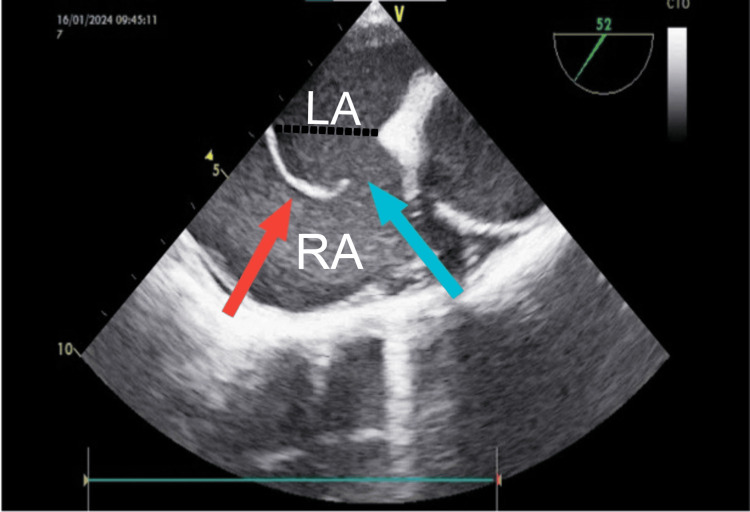
Transesophageal echocardiographic (TEE) image showing bulging atrial septum (red arrow) and the atrial septal defect (blue arrow). Black dotted line shows the imaginary septal plane. RA: right atrium; LA: left atrium.

**Figure 2:**
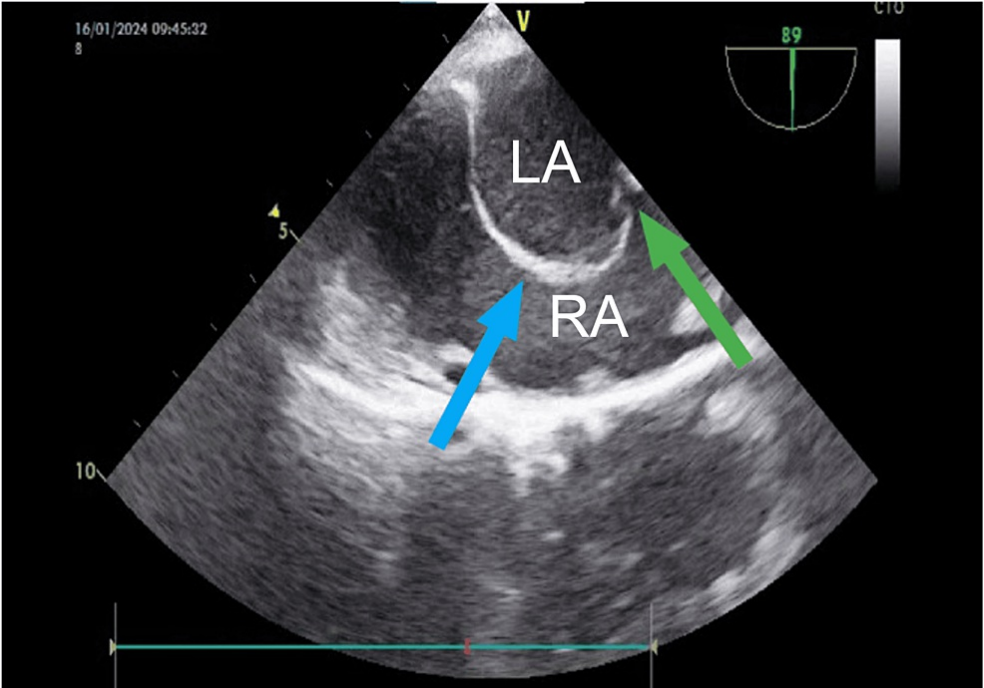
Transesophageal echocardiographic (TEE) image showing bulging atrial septum (blue arrow) and multiple fenestrations (green arrow). RA: right atrium; LA: left atrium.

**Figure 3:**
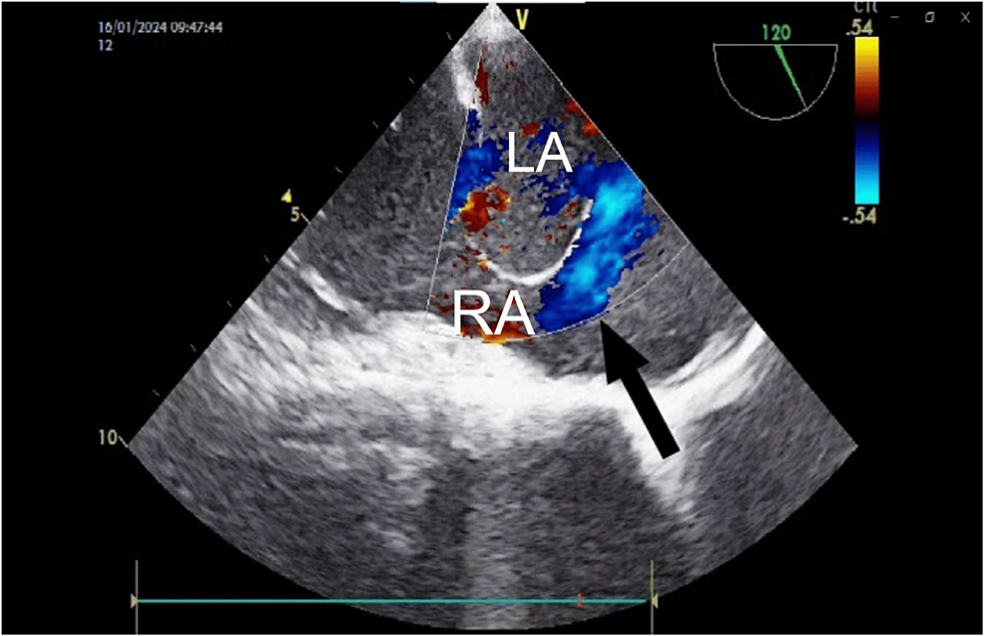
Transesophageal echocardiographic (TEE) image showing shunting across the atrial septal defect (black arrow). RA: right atrium; LA: left atrium.

On the day of the surgery, in the preoperative area, an 18-gauge peripheral line was secured in the patient's right hand. The patient was then wheeled into the operation theater, and monitors were attached according to the American Society of Anesthesiologists standards, which include electrocardiography, pulse oximetry, temperature monitoring, and non-invasive blood pressure monitoring. An arterial cannula was inserted in the left radial artery under local infiltration with 2% lignocaine. General anesthesia was administered with titrated doses of fentanyl (40 mcg) and a sleep dose of propofol (40 mg). The patient was intubated with a 7.5 mm internal diameter PVC cuffed endotracheal tube. A 7.5 French (Fr.) triple lumen central venous cannula (Multicath 3 UP, Vygon, Ecouen) was inserted into the right internal jugular vein. Anesthesia was maintained with 50% oxygen, 50% air, and isoflurane with a VMACage of 0.6-0.7. Muscle relaxation and analgesia were maintained with an infusion of vecuronium and fentanyl at doses of 1 mg/hour and 10 mcg/hour, respectively. After inserting a bite block, a transesophageal echocardiography (TEE) probe (GE 6Tc-RS cardiac echocardiography probe) was inserted. Examination revealed findings similar to the TTE (i.e., aneurysmal ASD with an excursion of 1.7 cm into the right atrium from the septal plane with multiple fenestrations, Type 1R as per Olivares-Reyes's classification of atrial septal aneurysm (ASA)). Below are the intraoperative TEE images showing the bulging atrial septum and the ASD (Figures 1-3). 

Below is the transesophageal echocardiographic video showing bulging atrial septum with the defect (Video 1).

**Video 1:** Transesophageal echocardiographic video showing aneurysmal atrial septum with the defect. View: mid-esophageal bicaval view.

Below is the transesophageal echocardiographic video showing aneurysmal ASD with multiple fenestrations (Video 2).

**Video 2:** Transesophageal echocardiographic video showing aneurysmal atrial septal defect with multiple fenestrations. View: mid-esophageal modified bicaval view.

Below is the transesophageal echocardiographic video showing aneurysmal ASD with multiple fenestrations. Color flow Doppler showing shunting across the defect and the fenestrations (Video 3).

**Video 3:** Transesophageal echocardiographic video showing aneurysmal atrial septal defect with multiple fenestrations. Color flow Doppler shows shunting across the septal defect and the multiple fenestrations. View: mid-esophageal modified bicaval view.

After achieving an adequate activated clotting time (ACT) of 480 seconds with unfractionated heparin (3 mg/kg = 160 mg), cardiopulmonary bypass (CPB) was initiated. Perfusion pressures of around 60-65 mmHg and urine output of 2 mL/kg were maintained during the CPB period. Arterial blood gas (ABG) parameters were within acceptable limits. Post-closure of the ASD, the patient was gradually weaned off CPB in sinus rhythm, maintaining a mean arterial pressure (MAP) in the range of 65-75 mmHg without any inotropic support. TEE performed after the ASD closure did not show any residual shunt. After completing the surgery, under ultrasound guidance, a bilateral pectointercostal fascial block (PIFB) was administered using 0.25% bupivacaine with a total volume of 30 mL. The patient was then shifted to the postoperative recovery room, where she was observed for one hour, maintaining stable hemodynamic and arterial blood gas levels within normal limits. According to the Enhanced Recovery After Surgery (ERAS) protocol in cardiac surgeries, the patient was extubated one hour post-surgery in the intensive care unit (ICU). The patient had an uneventful stay in the cardiac ICU post-surgery and was discharged from the hospital on postoperative day 7.

## Discussion

An ASD creates a left-to-right shunt, as the pressure in the right atrium is significantly lower than in the left atrium. The presentation and symptomatology of the patient depend on the size of the defect. Smaller defects (<5 mm) may not cause any symptoms, while larger defects may present with dyspnea, fatigue, and exercise intolerance [4]. Patients with small defects might experience spontaneous closure of the defect early in life, whereas larger defects require medical or surgical intervention [5].

The anesthetic management of an aneurysmal ASD includes decreasing the shunt fraction while simultaneously preventing the development of a hypoxic shunt phenomenon (right-to-left shunt). Both sedation and anesthesia are likely to affect changes in two parameters: systemic vascular resistance (SVR) and pulmonary vascular resistance (PVR), which are the primary factors influencing how the shunt behaves. Heart rate, contractility, and other characteristics are not as significant, as they do not influence the dynamics of the flow in the same manner as SVR and PVR. Avoiding medications that cause sustained increases in SVR is necessary, as it will amplify the left-to-right shunting. Similarly, prolonged exposure to a high inspired concentration of oxygen (FiO2) can also be detrimental as it lowers PVR and favors left-to-right shunting. A small decrease in SVR is desirable because it will decrease the fraction of the left-to-right shunt [6].

While ASDs are relatively common congenital defects, the presence of an aneurysmal septum adds complexity to the clinical presentation and management of the condition.

To diagnose an ASA, the atrial septum or a portion of it must show aneurysmal dilation protruding at least 1.5 cm beyond the atrial septum's plane, or phasic excursion during the cardiorespiratory cycle exceeding 1.5 cm, and the base of the aneurysmal protrusion must have a diameter of at least 1.5 cm [7]. The aneurysmal septum may result from abnormal tissue formation or remodeling processes during embryogenesis, leading to a weakened area in the atrial septum susceptible to dilation. An ASA may occur in association with other congenital valvular/septal defects or may exist by itself [8].

The association of an ASD with an ASA is uncommon, as shown in the study by Ruiz de Larrea [9], in which only 12 out of 5,221 cases were reported to have this. This increases the chances of cardioembolic stroke. Furthermore, the presence of an aneurysmal septum can complicate the diagnostic evaluation of ASDs, necessitating careful assessment with imaging modalities such as echocardiography, cardiac magnetic resonance imaging (MRI), or cardiac computed tomography (CT) to accurately characterize the extent and morphology of the defect.

Aneurysmal ASDs are categorized into five distinct types based on the direction and extent of the septum's bulging [10]. Type 1R features a bulge into the right atrium, while Type 2L bulges into the left atrium. Type 3RL is characterized by a significant bulge into the right atrium with a smaller bulge toward the left atrium. Conversely, Type 4LR exhibits a prominent bulge into the left atrium with a smaller deviation into the right atrium. Lastly, Type 5 involves bidirectional movement of the septum, with equal distance to both the right and left atria.

When compared to typical ASDs, aneurysmal ASDs are associated with a notably higher rate of complications, such as cardioembolic stroke, arrhythmias, and pulmonary hypertension [11].

In contrast to typical ASDs, aneurysmal ASDs present unique diagnostic challenges due to their dynamic nature and varying degrees of severity. Specialized imaging techniques, such as transesophageal echocardiography (TEE) or cardiac MRI, are necessary to assess the size, shape, and location of the ASD. Transesophageal echocardiography is superior to TTE in detecting an aneurysmal ASD, as in around 47% of the cases the aneurysmal ASD was missed on TTE [12].

The management of patients with aneurysmal ASDs also poses unique challenges. While device closure is a common option for managing typical ASDs, it may not be suitable for aneurysmal ASDs. The presence of an aneurysm may influence the selection of closure devices and procedural techniques. Multiple fenestrated ASDs may require the insertion of more than one device for closure [13]. Surgical intervention may be necessary to address the aneurysm and achieve optimal closure.

## Conclusions

Effective management of anesthesia for aneurysmal ASD requires a comprehensive understanding of its unique pathophysiological characteristics and associated challenges. By tailoring approaches to address hemodynamic instability, the risk of paradoxical embolism, and potential surgical complications, anesthesiologists can optimize patient outcomes and ensure perioperative safety. Collaboration among multidisciplinary teams, comprising cardiologists, surgeons, and anesthesiologists, is crucial for delivering holistic care and mitigating risks inherent to this intricate congenital anomaly. With ongoing advancements in perioperative monitoring, pharmacotherapy, and surgical methodologies, the management and prognosis of individuals with aneurysmal ASDs can continually be enhanced.
